# Supplementary material for: Splice-Junction-Based Mapping of Alternative Isoforms in the Human Proteome
Source: Cell Rep. Author manuscript; Available in PMC 2020 Jan 15. (PMC6961840; doi:10.1016/j.celrep.2019.11.026)
Supplement: 3 [file NIHMS1546469-supplement-3.zip › DF2/PXD009737/TestisLysC-119-O75608-YICPHAFDIIGLSPDSQEDESGIK.pdf]

A

Predicted sequence disorder and sequence features of O75608

Peptide: YICPHAFDIIGLSPDSQEDESGIK Junction: sp|O75608|LYPA1_HUMAN|ENSG00000120992|SE2|23679|chr8|54062324|54063375|−1|r4|T1 TrNovel: FALSE

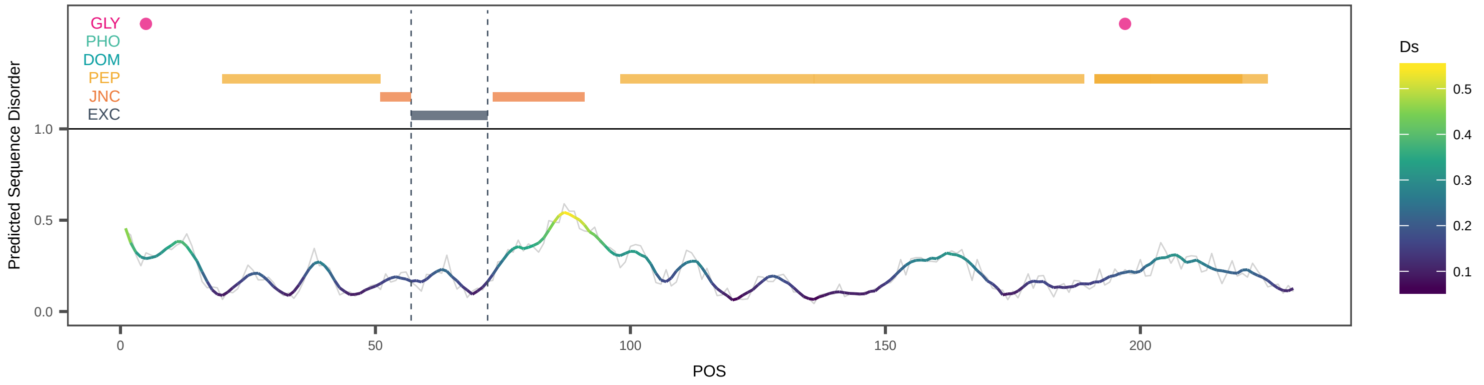

B

Distribution of sequence disorder in excised vs. mapped and non−excised regions of protein

M−W P−value vs. mapped: 0.424 vs. non−excised: 0.0848

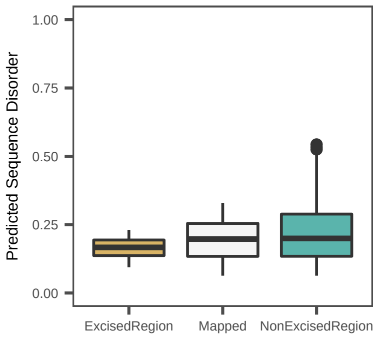

C
